# In Water Thermal Imaging Comparison of the Alcon Legacy and AMO Sovereign Phacoemulsification Systems§

**DOI:** 10.2174/1874364100802010020

**Published:** 2008-02-15

**Authors:** Kevin M Miller, Michael D Olson

**Affiliations:** Jules Stein Eye Institute and the Department of Ophthalmology, David Geffen School of Medicine at UCLA, Los Angeles, California, USA

## Abstract

**Purpose:**

To compare the temperature profiles of 2 popular phacoemulsification units under similar operating conditions in water.

**Methods:**

The phacoemulsification probes of the Sovereign WhiteStar and Legacy AdvanTec were capped with water-filled test chambers and imaged side-by-side using a thermal camera. The highest temperature of each chamber was measured at several time points after power application. Testing was performed under conditions capable of producing a corneal burn. The Legacy was operated in pulse mode at 15 Hz; a 50% duty cycle; and console power settings of 10, 30, 50 and 100%. The Sovereign was operated at the same console settings in WhiteStar C/F pulse mode at 56 Hz and a 33% duty cycle.

**Results:**

Under all conditions (powers of 10, 30, 50 and 100%; with or without irrigation/aspiration flow; and with or without sleeve compression), the Sovereign generated higher temperatures than the Legacy. At irrigation/aspiration flow rates ≥ 5 cc/min, the temperature profiles of the 2 units were indistinguishable.

**Conclusion:**

The Sovereign WhiteStar ran hotter than the Legacy AdvanTec under a variety of controlled low flow operating conditions. The Sovereign WhiteStar is more likely than the Legacy AdvanTec to produce a corneal burn under low flow conditions.

## INTRODUCTION

Thermal corneal injury is a known complication of ultrasonic phacoemulsification, and one of the reasons for the interest in alternative cataract removal technologies [1]. Corneal whitening, tissue coagulation, wound gape, wound closure difficulty, and high amounts of postoperative corneal astigmatism characterize severe thermal corneal injury. Corneal burns may be subtle as well; in some cases exhibiting only a curvilinear lucency and some faint whitening within the incision [2].

Corneal thermal injury is most likely to occur when a dense brunescent or black cataract is emulsified using high ultrasound energy and prolonged ultrasound time [3]. A burn is also more likely to occur when irrigation inflow is interrupted by a tight incision, or when outflow is interrupted by viscoelastic or cataract fragments in the lumen of the needle.

The cutting efficiency of phacoemulsifiers improves with each new generation. Older instruments have a greater propensity to generate heat and cause thermal injury than newer instruments [4]. Even within same-generation instruments, one manufacturer’s phacoemulsifier may generate more or less heat than another manufacturer’s instrument. For example, Mackool and Sirota recently found that the Advanced Medical Optics Sovereign WhiteStar and the Bausch & Lomb Millennium generate higher peak temperatures than the Alcon Legacy AdvanTec when operated under identical console power and duty cycle settings [5]. Even phacoemulsification needle design and the angle of incision entry can affect thermal performance [6].

Previously we performed an in-air thermal imaging study using the same 3 phacoemulsifiers as Mackool and Sirota. We operated the hand pieces side by side in air to maintain absolute control over the flow of irrigating fluid. Not surprisingly, we came to the same conclusion as Mackool and Sirota, which is the Legacy AdvanTec ran cooler than the other two phacoemulsifiers [7]. As a follow-up study, we felt it would be appropriate to run the experiments again in fluid-filled chambers to simulate the fluidic environment of the human eye.

In this series of experiments we ran the Sovereign WhiteStar and the Legacy AdvanTec side by side in water-filled plastic test chambers and measured the peak temperatures using the same thermal imaging camera. This time we did not test the Millennium.

## METHODS

**Fig. (1):**
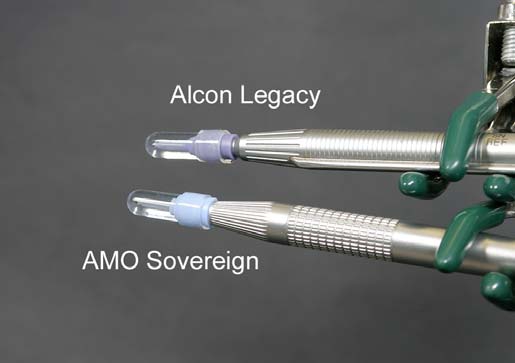
Phacoemulsification probes from the Alcon Legacy and AMO Sovereign were capped with water-filled silicone test sleeves, which functioned as calorimeters for these experiments.

The probes of the Alcon Legacy AdvanTec and AMO Sovereign WhiteStar phacoemulsification units were capped with water-filled test chambers (volume 0.85cc, approximately 4 times greater than the anterior chamber of the human eye) and imaged side-by-side in the infrared using an FLIR model P60 ThermaCAM™ (FLIR Systems Inc., North Billerca, MA). The probes were placed in a common plane perpendicular to the camera so they would be simultaneously in focus (Fig. **1**). This camera is capable of measuring temperatures over a dynamic range of –40°F to +248°F (-40°C to 120°C). For these experiments, the camera was set to display linearly between 65°F and 130°F (18°C and 55°C). Camera calibration was verified by measuring hot and cold-water baths using both the infrared camera and a thermocouple thermometer at temperatures across the experimental range.

**Fig. (2):**
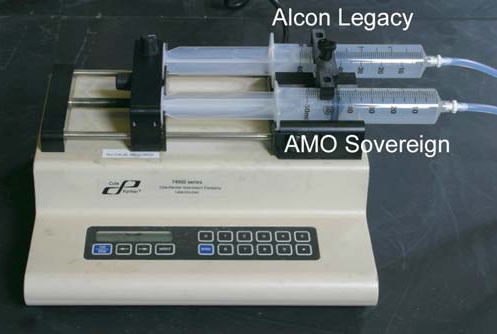
Controlled irrigation flow was delivered to the phacoemulsification probes using a Cole Palmer dual syringe pump.

The flow conditions were designed to simulate those under which a corneal burn might develop. Irrigation/aspiration flow was set at 0 cc/min in some experiments to simulate complete occlusion of the tip, and at 1 cc/min in other experiments to simulate incomplete occlusion of the tip. Aspiration flow pumps on all modern phacoemulsification units cannot be programmed for low flow rates, so instead a Cole Palmer 74900 Series duel syringe pump was used to control flow (Fig. **2**). There was no leakage from the systems, so irrigation and aspiration flow rates were identical. Bottle height was 21 inches above probe height in the zero flow experiments. In each experiment, 0.9 mm phacoemulsification needles with 30° bevels (Alcon Legacy RD 8065-740837; AMO Sovereign T25930) were used. The distal tips of the silicone sleeves were set back 1 mm from the tips of the needles. The side ports on the silicone sleeves were oriented 90° from the beveled surfaces of the needles.

We ran 5 experiments in this study, applying or removing irrigation/aspiration flow and external weights between experiments. We operated the 2 phacoemulsifiers in pulse mode in all experiments. The Alcon Legacy AdvanTec was programmed for 15 Hz (the maximum pulse frequency available). The Legacy has a fixed “on” time of 50% of the duty cycle. The AMO Sovereign was operated in WhiteStar C/F mode. This should give it a thermal advantage over the Legacy, all other things being equal, because power is applied only 33% of the time in C/F mode. The Sovereign pulse rate is fixed at 56 Hz in C/F mode. These frequency and duty cycle settings are common in clinical practice.

**Fig. (3):**
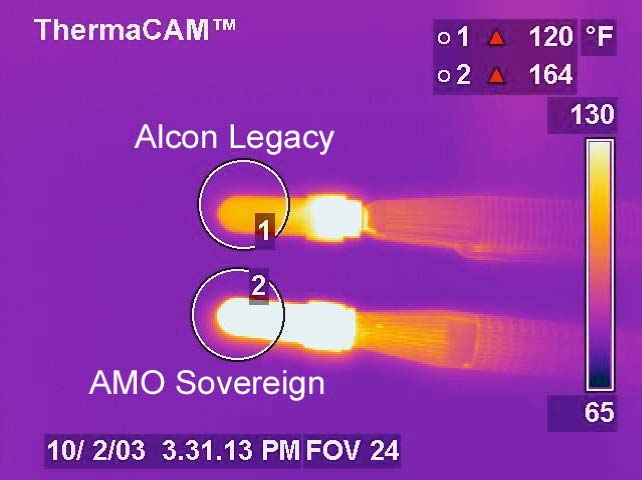
This digital thermal camera image of the phacoemulsification probes and test sleeves was obtained 120 seconds after power application in Experiment 1. The Alcon Legacy probe, operating in pulse mode at 100% power, achieved a peak temperature of 120°F. The AMO Sovereign probe, operating in WhiteStar C/F pulse mode at 100% power, achieved a peak temperature of 164°F. These data points are bar graphed in the bottom panel of Fig. **(5)**.

The FLIR camera captured still images of the probes before power application (at 0 seconds), and then 10, 30, 60 and 120 seconds after power application. Experiments were allowed to run for 2 minutes because the large volume of fluid in the test chambers takes significantly longer to heat than the aqueous humor in the human eye. The test chambers function like calorimeters except that there is some heat transfer along the phacoemulsification hand pieces. Digital images were saved to disk at each time point (Fig. **3**). The highest surface temperature inside a circle drawn around each water-filled test chamber was recorded. All temperatures were rounded to the nearest whole°F. The temperatures were graphed as a function of time and applied power, grouped by phacoemulsification unit.

### Experiment 1

Phacoemulsification power on both instrument consoles was set at 10%, 30%, 50% and 100% in 4 separate measurements. The highest surface temperature of each probe was recorded at 0, 10, 30, 60 and 120 seconds. Irrigation/aspiration flow was set at 0 cc/min to simulate complete occlusion of the tip.

### Experiment 2

**Fig. (4):**
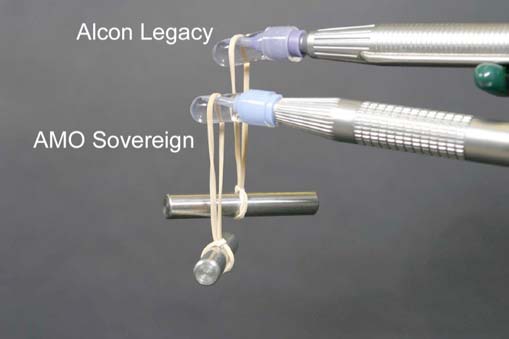
We increased friction between the phacoemulsification needle and sleeve in experiments 2 and 4 by suspending 19.9 gm weights from the test chambers. (The results of experiments involving sleeve compression are shown in Figs. **(6,8)**.

19.9-gm weights were suspended from the water-filled test chambers to simulate the increased friction produced by a tight corneal incision (Fig. **4**). The rubber bands suspending the weights squeezed the test chamber walls against the silicone sleeves, increasing the friction between the silicone sleeves and the phacoemulsification needles. All other parameters were the same as in experiment 1.

### Experiment 3

Irrigation/aspiration flow was set at the low rate of 1 cc/min to simulate incomplete tip occlusion. The weights were removed, so the silicone sleeves were not compressed. All other parameters were the same as in experiment 1.

### Experiment 4

Irrigation/aspiration flow was set at the low rate of 1 cc/min to simulate incomplete tip occlusion. The water-filled test chambers were compressed by suspending 19.9-gm weights. The parameters were otherwise the same as in experiment 1.

### Experiment 5

Irrigation/aspiration flows were set at 0, 1, 5, 10 and 30 cc/min in 5 separate measurements. Phacoemulsification power was set at 100% on both instruments. The highest surface temperatures were recorded 10, 30, 60 and 120 seconds after power application.

## RESULTS

The results of the 5 experiments are shown in Figs. (**5-9**). The data are grouped by phacoemulsification unit. Each graph in a stack represents a single point in time. The temperatures recorded in the top graphs were taken at time 0 (room temprature). Temperatures in the middle 3 graphs were taken 10, 30 and 60 seconds after power application, and those in the bottom graphs were taken at 120 seconds. The graphs in Figs. (**5-8**) show cumulative data for separate runs at powers of 10%, 30%, 50% and 100%. The graphs in Fig. (**9**) show data from 5 separate experiments performed at 100% power and irrigation flow rates of 0, 1, 5, 10 and 30 cc/min.

Each data point or bar in a graph represents the highest temperature recorded over the surface of a test chamber. To reach this temperature the phacoemulsification probe had to heat the silicone test chamber and the entire volume of water contained within it (calorimeter). Under clinical conditions, a corneal burn would occur much faster than under these experimental conditions.

### Experiment 1

**Fig. (5):**
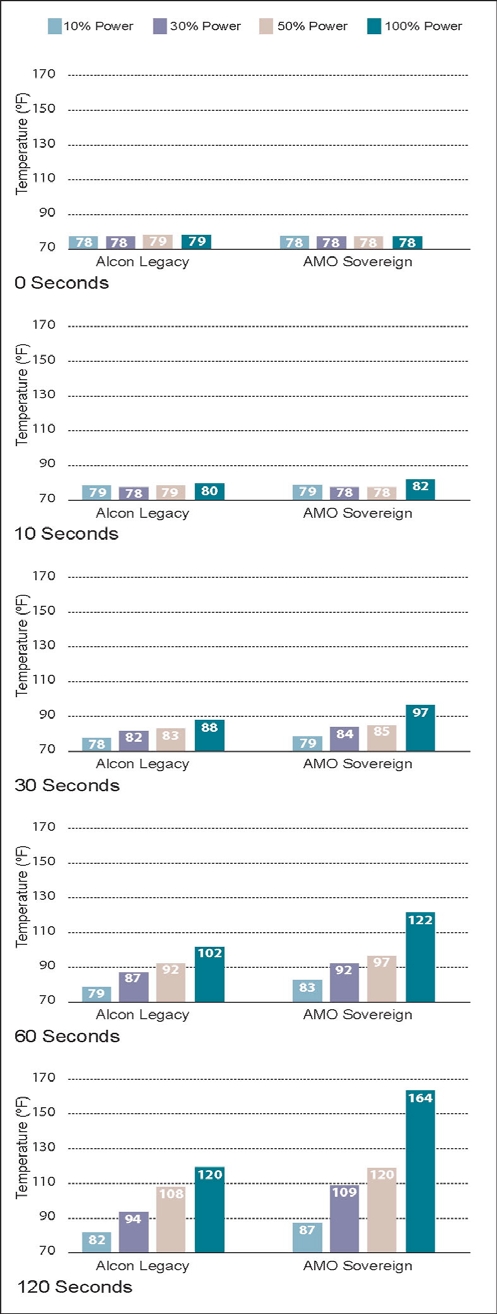
This chart shows phacoemulsification probe temperature in °F as a function of console power and time as described in Experiment 1. Irrigation/aspiration flow was set to zero and no weights were suspended from the test chambers in this experiment.

With no irrigation/aspiration flow, the Sovereign ran hotter than the Legacy at the 10%, 30%, 50% and 100% power settings (Fig. **5**). The Sovereign achieved the highest temperature (164°F).

### Experiment 2

**Fig. (6):**
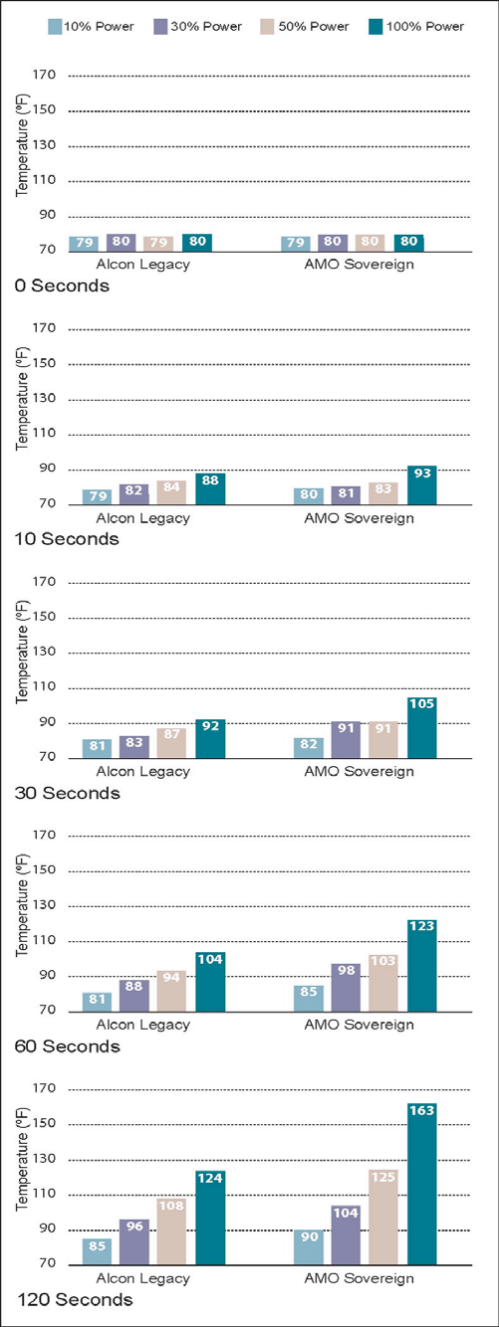
This chart shows phacoemulsification probe temperature in °F as a function of console power and time as described in Experiment 2. Irrigation/aspiration flow was set to zero and 19.9 gm weights were suspended from the test chambers in this experiment.

With 19.9-gm weights hanging from the water-filled test chambers and no irrigation/aspiration flow, the Sovereign ran hotter than the Legacy at the 10%, 30%, 50% and 100% power settings (Fig. **6**). The Sovereign achieved the highest temperature (163°F).

### Experiment 3

**Fig. (7):**
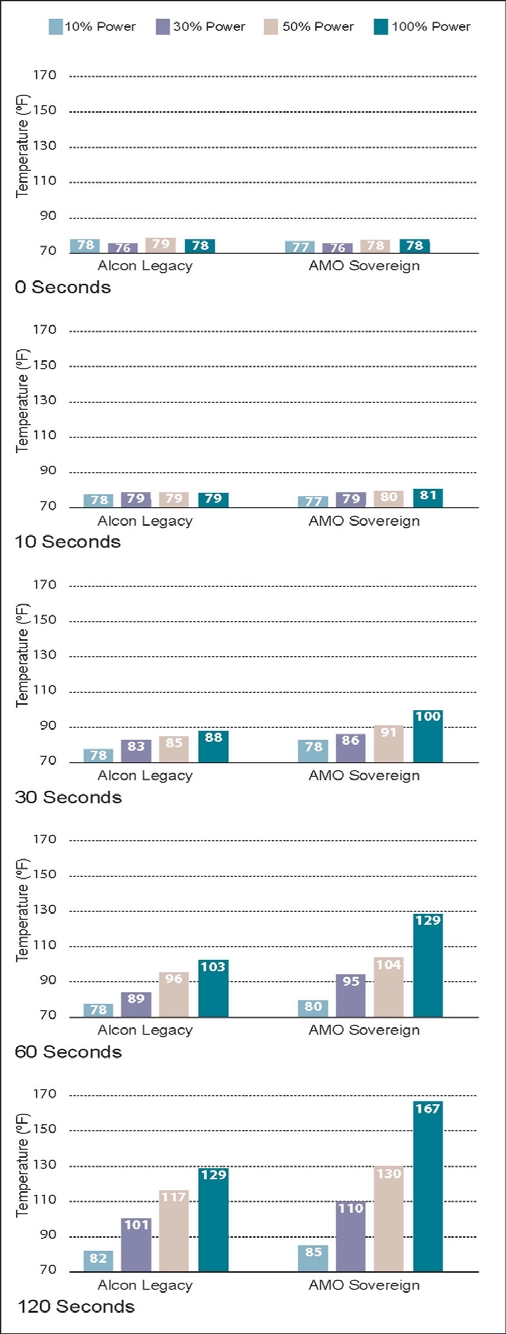
This chart shows phacoemulsification probe temperature in °F as a function of console power and time as described in Experiment 3. Irrigation/aspiration flow was set to 1 cc/min and no weights were suspended from the test chambers in this experiment.

With irrigation/aspiration flow set at 1cc/min to simulate partial occlusion of the tip, the Sovereign ran hotter than the Legacy at the 10%, 30%, 50% and 100% power settings (Fig. 7). The Sovereign achieved the highest temperature (167°F). Interestingly, both machines achieved higher peak temperatures when there was low flow as compared to no flow.

### Experiment 4

**Fig. (8):**
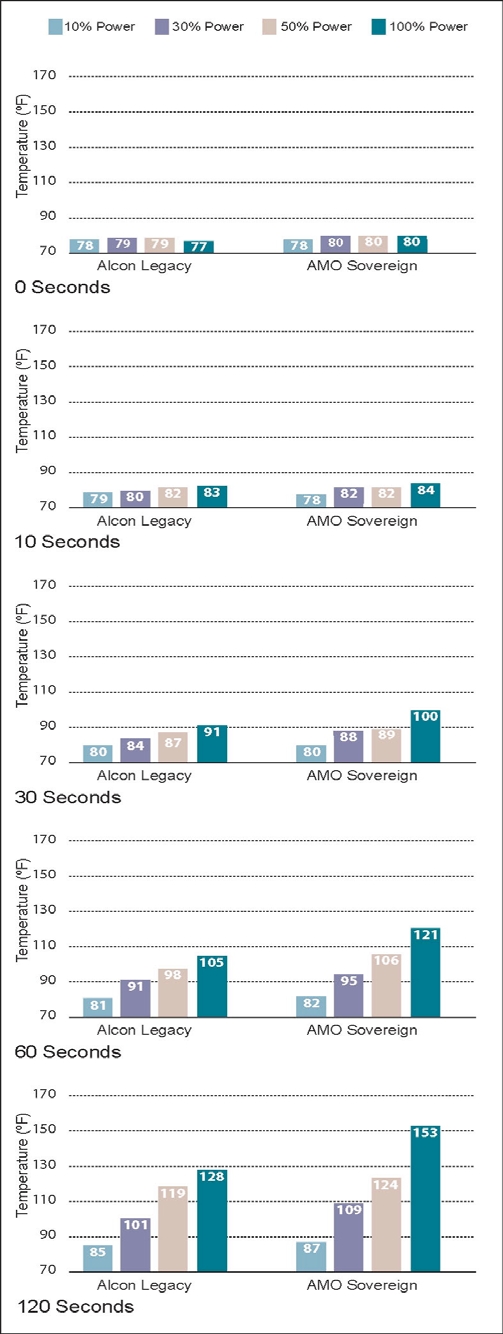
This chart shows phacoemulsification probe temperature in °F as a function of console power and time as described in Experiment 4. Irrigation/aspiration flow was set to 1 cc/min and 19.9 gm weights were suspended from the test chambers in this experiment.

With 19.9-gm weights hanging from the water-filled test chambers and irrigation/aspiration flow set at 1cc/min, the Sovereign ran hotter than the Legacy at the 10%, 30%, 50%, and 100% power settings (Fig. **8**). The Sovereign achieved the highest temperature (153°F).

### Experiment 5

**Fig. (9):**
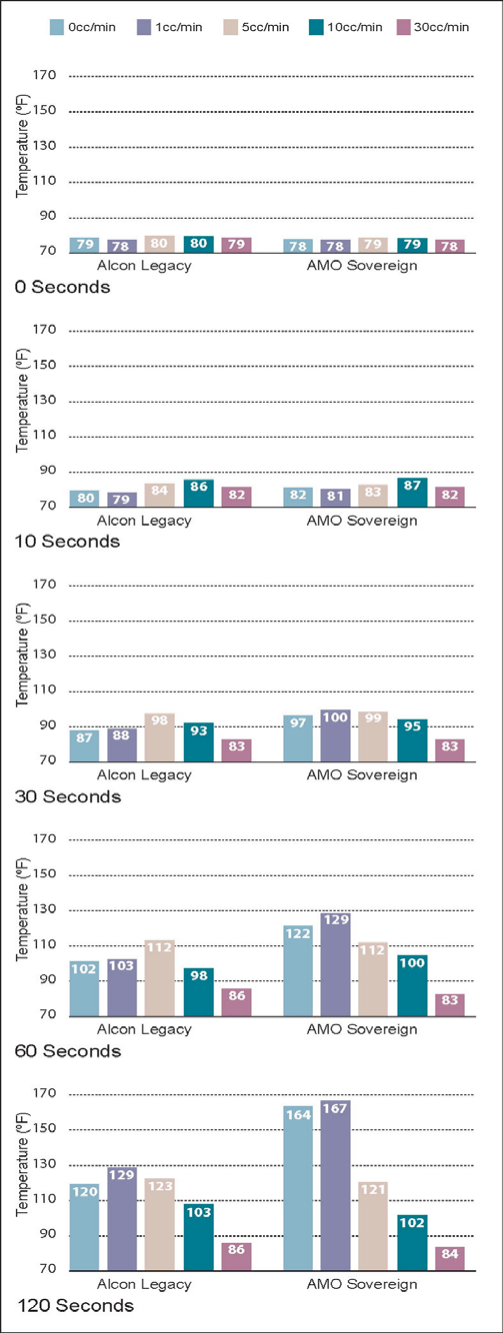
This chart shows phacoemulsification probe temperature in °F as a function of irrigation/aspiration flow and time as described in Experiment 5. Console power was held constant at 100%. No weights were suspended from the test chambers in this experiment.

This experiment tested the effect of irrigation/aspiration flow on peak temperatures. At flow rates of 0 and 1 cc/min, the Sovereign ran hotter than the Legacy. At flow rates of 5cc/min or more the 2 instruments were indistinguishable (Fig. **9**).

## DISCUSSION

Under all experimental conditions (powers of 10, 30, 50 and 100%; with or without irrigation/aspiration flow; and with or without sleeve compression), the AMO Sovereign WhiteStar generated higher temperatures than the Alcon Legacy AdvanTec. At irrigation/aspiration flow rates ≥ 5 cc/min, the temperature profiles of the 2 units were indistinguishable.

The water-filled test chambers utilized in this study allowed us to simulate the fluid environment of the human eye while maintaining absolute control over irrigation. In this respect, the experiments of this study are more clinically relevant than those of the previous study in which the probes were operated in air [7]. It is critically important that irrigation flow be controlled precisely whenever thermal studies are conducted. A small difference in irrigation flow produces a significant difference in results as Experiment 5 demonstrates. It is extremely difficult to control flow in human cadaver and animal eye experiments, and for this reason this model was not used. A disadvantage of using water-filled test chambers is that we cannot image the phacoemulsification needles directly. The infrared camera does not “see” the needles. Instead it “sees” and measures the temperature of the outside surface of the test chambers, which correlates to the temperature of the water and phacoemulsification needles inside the test chambers. Also, there is a greater latency for temperature rise using this experimental setup than there would be *in vivo*.

Operating at 50% power and an irrigation flow rate of 1 cc/min, both instruments achieved temperatures capable of producing corneal burns. An interesting observation is that the temperatures achieved with flow rates of 1 cc/min were slightly higher than with zero flow (Figs. **7**,**8**). We think this is the result of irrigating fluid preheating as it passes through the hand piece containing the piezoelectric crystals.

Under conditions of loading, the probes of both instruments experienced faster temperature rise times. The Alcon legacy achieved similar or slightly higher peak temperatures because it responds to loading by maintaining constant stroke. The AMO Sovereign achieved lower peak temperatures because it loses stroke under conditions of loading. Instead it maintains constant power.

This study was conducted in a Consumer Reports fashion. The assumption in this type of analysis is that devices or instruments from different manufacturers are more or less the same. While this is generally true (both instruments emulsify cataracts using piezoelectric crystal-generated ultrasound energy), there are differences. Industry has not agreed on a definition for 100% power, so the machines are different in this respect. The machines also differ in control circuitry—the Legacy is designed to maintain constant stroke under varying load conditions while the Sovereign is designed to deliver constant power to the piezoelectric crystal independent of load. These differences affect how the 2 machines function under conditions of variable loading. The Legacy maintains a constant stroke or cutting force (needle excursion) when subjected to varying loads, but the Sovereign loses stroke as load is increased. At the same control panel power setting, the Sovereign strokes more when it is not loaded (no weight suspended from the needle) than when it is loaded—this is partially why, on average, the Sovereign generated slightly more heat in our experiments when not loaded. Despite the potential criticism that these experiments compare apples to oranges because the machines function differently, the comparison is useful from a practical perspective because the average ophthalmologist who must decide between machines when making a purchase decision is not generally aware of the intricacies of instrument software and circuitry.

We would like to emphasize that the Legacy AdvanTec and Sovereign WhiteStar phacoemulsification units are indistinguishable from a thermal perspective when operated under conditions of no load or minimal load in the normal range of aspiration flow (>5cc/min). All phacoemulsifiers, regardless of manufacturer, should be operated with good irrigation/aspiration flow, maintaining the lowest power setting necessary to emulsify lens material efficiently.
